# Intrinsic capacity and self-perceived health among home-dwelling older adults: The HUNT study

**DOI:** 10.1016/j.tjfa.2026.100188

**Published:** 2026-09-04

**Authors:** Kjerstin Næss Melsæter, Turid Follestad, Gro Gujord Tangen, Beatrix Vereijken, Pernille Thingstad

**Affiliations:** a Department of Neuromedicine and Movement Science, Faculty of Medicine and Health Sciences, NTNU, Norwegian University of Science and Technology, 7491, Trondheim, Norway; b Trondheim Municipality, Trondheim, Norway; c Department of Public Health and Nursing, Faculty of Medicine and Health Sciences, NTNU, Norwegian University of Science and Technology, 7491, Trondheim, Norway; d The Norwegian National Centre for Ageing and Health, Vestfold Hospital Trust, Tønsberg, Norway; e Department of Geriatric Medicine, Oslo University Hospital, Oslo, Norway

**Keywords:** Intrinsic capacity, Self-perceived health, Locomotor capacity, Older adults, HUNT

## Abstract

**Purpose:**

To describe intrinsic capacity (IC) distribution across age and sex and investigate its subdomains’ association with self-perceived health in home-dwelling older adults.

**Methods:**

This cross-sectional study used population-based data from the Trøndelag Health Study (HUNT). Self-perceived health was assessed by a 4-point Likert scale and dichotomized into good or poor health. IC consists of five subdomains (vitality, locomotion, cognition, psychological, and sensory capacity), assessed in line with the WHO recommendations. Capacity in locomotion and cognition was assessed by the SPPB and MoCA, while the remaining subdomains were assessed through questionnaires. Associations between subdomains and self-perceived health were examined using simple and multiple regression models, adjusting for age and education.

**Results:**

We included 8718 home-dwelling older adults (aged 70–101 years; 52.5% women). Good health was reported by 69.8% among those < 85 years and by 48.6% among those ≥ 85 years. High capacity in ≥ 3 subdomains was observed in 79.3% and 40.9% among individuals < 85 years and ≥ 85 years, respectively. High locomotor capacity showed the strongest association with good self-perceived health. The probability of poor self-perceived health increased with lower IC, but among women, significantly less by older age and more by higher education.

**Conclusion:**

We observed a strong association between IC and self-perceived health, suggesting that IC is closely linked to individuals’ self-perceived health. Our findings support IC as a relevant tool in the shift toward preventive, health-promoting care, and we believe the use of IC can help tailor interventions to optimize functional ability and well-being.

## Highlights

• Intrinsic capacity was associated with self-perceived health in community-dwelling older adults.
• Locomotor capacity demonstrated the strongest association with self-perceived health.
• Reduced capacity in any subdomain was associated with an increased probability of poor self-perceived health, with the association moderated by age and education among women.

## 1 Introduction

With an aging population [1], understanding which factors contribute to healthy aging is increasingly important for providing sustainable healthcare [2,3]. The World Health Organization (WHO) has advocated for a shift in focus from disease management to identifying and supporting the determinants of health and quality of life. This perspective is operationalized and described within the Integrated Care for Older People (ICOPE) framework [1]. Central to this framework is the concept of intrinsic capacity (IC), defined as “the composite of all the physical and mental capacities that an individual can draw on” [1]. IC represents a positive and holistic view of aging, emphasizing individuals’ resources and potential [1,4,5].

Current evidence supports the five-subdomain structure of IC: vitality, locomotion, cognition, psychological capacity, and sensory (hearing and vision) capacity [[5], [6], [7]]. The ICOPE framework provides clinically accessible screening tools [6], with a clear emphasis on early risk detection and preventive interventions. IC is validated as a reliable measure of functional ability [8], examined in both clinical samples [[9], [10], [11]] and population-based cohorts [7,12,13]. However, many existing studies are limited by small (<1000 participants) [9,10,12,13], relatively young cohorts (<70 years old) [7,9,11,12,14,15], or by using IC measures not routinely available in clinical practice [7,15]. This provides potentially biased information about the range of functional abilities among community-dwelling older adults. Building population-level knowledge about IC in older adults with a broader range of age and function, using assessments easily applicable in practice, is critical to ensure clinical relevance and establish a strong foundation for preventive strategies in an aging population.

Despite current guidance on IC assessments [6], there is limited consensus on how IC should be operationalized [16], and previous studies have employed a wide range of assessments, many of which are impractical for routine clinical use [5,7,[16], [17], [18]]. Vitality shows the greatest variability across assessments, whereas the sensory domains are most often assessed through self-report [5]. The lack of standardization and the discrepancy between the ICOPE framework and existing research highlight the need for more studies using accessible, practice-oriented assessments.

Health is defined as “a state of complete physical, mental, and social well-being and not merely the absence of disease or infirmity” [19]. Consequently, the relevance of the ICOPE frameworks’ standardized IC measurements hinges on the assumption that they closely reflect older adults’ health perception. To our knowledge, few studies have explored this relationship in large, population-based samples, and little is known about how self-perceived health relates to specific IC subdomains. Thus, understanding this association is important for establishing IC as a comprehensive health measure in older adults. To address these identified gaps, the present study aims to describe the distribution of IC across age and sex in a population-based sample of community-dwelling older adults in Norway, and to examine the associations between IC subdomains and self-perceived health.

## 2 Methods

### 2.1 Study design and study population

This population-based, cross-sectional study is based on home-dwelling older participants in the fourth wave of the Trøndelag Health Study (HUNT4) 70+ (2017 – 2019) and the Trondheim 70+ sub-study (2018 – 2019) [[20], [21], [22]]. The HUNT Study has collected health data through questionnaires, biological samples, and clinical measurements across four waves since 1983 within the population of the former Nord-Trøndelag county [20,23], with an added focus on participants 70 years and older in HUNT4 (HUNT4 70+). To capture a more urban population, a district in Trondheim city was also included (Trondheim 70+). Participants completed questionnaires, interviews, and clinical assessments, either at a test station or in their own homes, assessed by an ambulatory team. Detailed protocols for HUNT4 70+ and Trondheim 70+ have been published previously [20,21]. For this study, the two cohorts were combined.

**Fig. 1:**
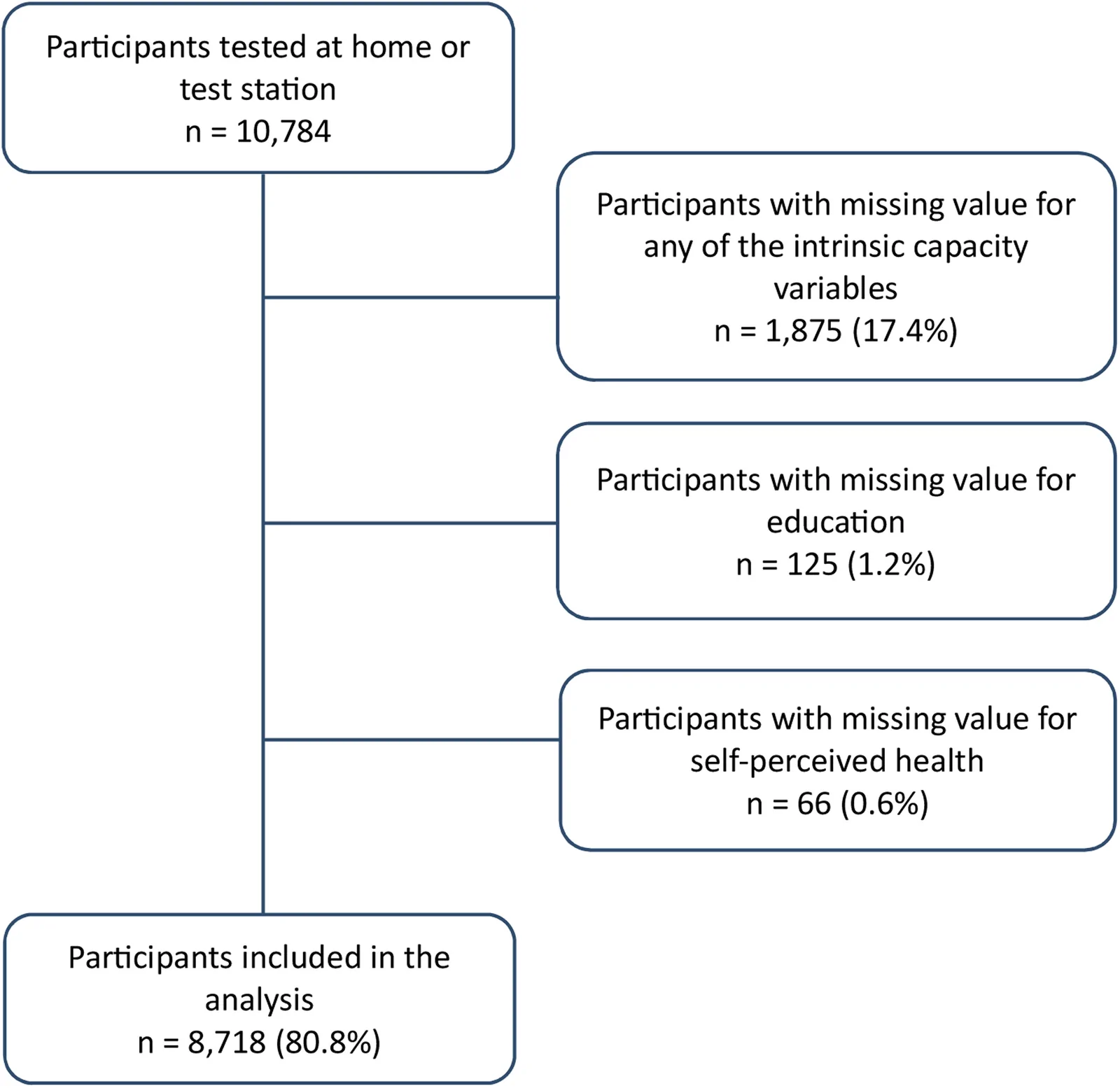
Flow chart.

In this study, we included home-dwelling individuals participating in HUNT4 70+ and Trondheim 70+ (n = 10,784). Those with missing values for any of the IC subdomains, education, or self-perceived health (n = 2066) were excluded, resulting in a total of 8718 (80.8%) participants (see Fig. 1). Excluded participants were older (80.4 (SD 7.2) versus 76.8 (SD 5.5)), had a higher proportion of participants with a Short Physical Performance Battery (SPPB) score below 10 (54.1% vs 25.7%), and a lower proportion reporting good health (51.7% vs 67.8%), compared to those included in this study. Additionally, a higher proportion of excluded participants were tested in their own homes (27.9% vs 4.5%).

### 2.2 Assessment of intrinsic capacity subdomains

*Vitality* was based on two questions: “Have you had an unintentional weight loss of more than 5 kg during the last 6 months?” and “Has your appetite been reduced during the last 4 weeks?”. Participants answering yes to either of these questions were categorized as having low vitality. *Locomotor* and *cognitive capacity* were assessed using performance-based tests. The SPPB was used to assess locomotor capacity, which consists of three timed subtests: a hierarchic static balance test, a normal pace 4-meter gait speed test, and a 5 times sit-to-stand test [24,25]. Based on time scores, participants were given a total score between 0 and 12 points, with a higher score indicating better physical performance. For the analysis, SPPB was converted into a dichotomous variable, classifying participants as having low locomotor capacity if their SPPB score was ≤ 9. Cognitive capacity was assessed through the Montreal Cognitive Assessment (MoCA) [26], a multidomain screening tool testing memory, visuospatial and executive functions, attention, and orientation. MoCA is scored between 0 and 30 points, with a higher score indicating better cognitive function [22]. Using findings from a prior HUNT study [26], we created a dichotomous variable based on normative MoCA scores adjusted for age, sex, and education, using the −1.5 standard deviation (SD) as a cut-point. For participants with missing data on education, we used a MoCA score below 22 points to categorize participants with low cognitive capacity [9]. *Psychological capacity* was based on the Hospital Anxiety and Depression Scale (HADS) total score and a question about depressive mood. HADS is scored between 0 and 21 points, where a higher score indicates more severe symptoms [27]. Participants with > 7 points on HADS or answering “yes” to the question “Have you for the last 2 weeks felt down/depressed?” were considered to have low psychological capacity. *Hearing* and *visual capacity* were based on a yes/no question: “Do you suffer from a longstanding (at least 1 year) illness or injury of a physical or psychological nature that impairs your functioning in your daily life?” followed by a checklist for different conditions, including vision and hearing. Those answering yes to visual or hearing impairments were classified as having low capacity within the respective subdomain.

In line with a recently published review [28], we classified participants based on the distribution of low versus high capacity across the IC subdomains: high IC (high capacity in all subdomains), intermediate IC (low capacity in 1 or 2 subdomains), and low IC (low capacity in 3 or more subdomains).

### 2.3 Self-perceived health

Self-perceived health was based on a 4-point Likert scale (poor, not so good, good, and very good) question: “How is your health at the moment?”. For the analysis, self-perceived health was converted into a dichotomous variable combining “good” and “very good” into good self-perceived health, and “poor” and “not so good” into poor self-perceived health.

### 2.4 Statistical analysis

All analyses were performed using STATA Statistical Software version 18. Depending on the distribution, we used mean and standard deviation (SD), or frequency and percentage, to summarize the participants' characteristics and to describe the distribution of the IC subdomains across age and sex. To estimate the association between IC and self-perceived health, we used logistic regression models and performed three types of analyses for each IC subdomain: an univariable analysis (Model 1), an analysis adjusted for sex, age, and education (Model 2), and an analysis adjusted for sex, age, education, and all remaining IC subdomains (Model 3). A p-value < 0.01 was set to define statistical significance. Finally, we fitted a model for the association between the total number of IC subdomains and self-perceived health, adjusting for age and education and including two-way and three-way interaction terms between the number of IC subdomains, age, and education. The number of IC subdomains was treated as a continuous variable in this model. To assess whether the inclusion of an interaction term improved model fit, we compared two nested logistic regression models, one with and one without the interaction term, using a likelihood ratio test. Only significant interaction terms were kept in the models for simplicity and interpretability. The regression models were fitted for the total study sample with sex as a covariate, and for men and women separately.

## 3 Results

The 8718 participants included in the analysis (52.5% women) were aged 70 to 101 years. A total of 67.8% reported good health, and 32.6% were living alone. Relevant characteristics of the participants are presented in Table 1.

**Table 1:** Sample characteristics by age group and sex (n = 8718).

Age group	Total	70-74		75-79		80-84		85-89		90+	
Sex		women *(n = 2094)*	men *(n = 2002)*	women *(n = 1289)*	men *(n = 1183)*	women *(n = 704)*	men *(n = 627)*	women *(n = 346)*	men *(n = 235)*	women (*n = 140)*	men *(n = 98)*
Home visit, n(%)	397 (4.5)	21 (1.0)	14 (0.7)	32 (2.5)	28 (2.4)	55 (7.8)	21 (3.4)	78 (22.5)	32 (13.6)	81 (57.9)	35 (35.7)
Level of education, n(%)											
≤ 10 years	2,144 (24.6)	435 (20.8)	278 (13.9)	414 (32.1)	207 (17.5)	302 (42.9)	150 (23.9)	177 (51.2)	59 (25.1)	93 (66.4)	29 (29.8)
11–13 years	3,881 (44.5)	1,021 (48.7)	907 (45.3)	555 (43.1)	546 (46.2)	251 (35.8)	285 (45.5)	114 (32.9)	115 (48.9)	36 (25.7)	51 (52.0)
≥ 14 years	2,693 (30.9)	638 (30.5)	817 (40.8)	320 (24.8)	430 (36.3)	151 (21.5)	192 (30.6)	55 (15.9)	61 (26.0)	11 (7.9)	18 (18.4)
Living alone, n(%)	2,801 (32.6)	632 (30.5)	316 (16.0)	543 (43.0)	211 (18.0)	431 (62.9)	145 (23.5)	269 (79.8)	89 (38.7)	120 (91.6)	45 (46.4)
Self-reported health, n(%)											
Good or very good	5,909 (67.8)	1,510 (72.1)	1,517 (75.8)	875 (67.9)	815 (68.9)	392 (55.7)	402 (64.1)	173 (50.0)	121 (51.5)	56 (40.0)	48 (49.0)
Weight loss, n(%) (n = 8,120)	382 (4.7)	84 (4.3)	74 (3.9)	45 (3.8)	48 (4.3)	50 (7.9)	26 (4.5)	24 (8.1)	12 (5.5)	11 (8.7)	8 (9.1)
Reduced appetite, n(%) (n = 8,697)	608 (7.0)	147 (7.0)	87 (4.4)	97 (7.5)	61 (5.2)	70 (10.0)	28 (4.5)	46 (13.3)	30 (12.8)	25 (17.9)	17 (17.5)
SPPB^a^, mean (SD)	10.1 (2.5)	10.7 (1.9)	11.0 (1.7)	10.2 (2.2)	10.5 (2.1)	9.0 (2.9)	9.8 (2.5)	7.4 (3.1)	7.9 (3.3)	5.0 (3.1)	6.0 (3.4)
MoCA^b^, mean (SD)	23.4 (4.1)	24.8 (3.2)	23.9 (3.3)	24.0 (3.6)	23.3 (3.7)	22.4 (4.4)	21.9 (4.0)	20.0 (5.0)	20.6 (4.4)	17.0 (6.1)	18.1 (5.4)
HADS^c^, mean (SD) (n = 6,660)	3.5 (2.8)	3.0 (2.6)	3.4 (2.7)	3.3 (2.5)	3.7 (2.8)	4.0 (2.7)	4.4 (3.0)	4.3 (3.0)	4.8 (3.2)	4.9 (3.0)	5.1 (3.2)
Feel down/depressed, n(%)	1,777 (21.7)	479 (24.4)	307 (15.8)	271 (23.2)	207 (18.4)	186 (29.4)	112 (19.0)	93 (29.3)	59 (27.2)	38 (29.2)	25 (27.5)

a SPPB: 0–12 points, higher score indicates better performance.

b MoCA: 0–30, higher score indicates better performance.

c HADS: 0–21, higher score indicates more severe symptoms.

Among participants younger than 85 years old, 69.8% reported good health and 79.3% had high or intermediate IC, compared to 48.6% and 40.9%, respectively, in participants 85 years and older. We observed an age-related difference in high capacity, with the highest percentage in the youngest age group and the lowest in the oldest. The most prominent differences were observed in locomotor capacity (Table 2). Within both locomotor and visual capacity, high capacity was most pronounced among men, while high cognitive and hearing capacity were more prevalent in women, except for the oldest age cohorts (Table 2). The overall highest percentage of participants with high capacity was observed in vitality, with 90.1% (women) and 92.6% (men) among the youngest, and 78.6% (women) and 77.5% (men) among the oldest participants.

**Table 2:** Percentage (%) of participants with high capacity in each intrinsic capacity (IC) subdomain by age group and sex (n = 8718).

Age group	Total	70–74		75–79		80–84		85–89		90+	
Sex		women *(n=2094)*	men *(n=2002)*	women *(n=1289)*	men *(n=1183)*	women *(n=704)*	men *(n=627)*	women *(n=346)*	men *(n=235)*	women (*n=140)*	men *(n=98)*
Vitality	89.7	90.1	92.6	89.8	91.6	84.9	92.3	82.1	83.8	78.6	77.5
Locomotion	74.3	84.8	88.4	74.6	80.3	52.3	67.5	29.5	40.8	8.6	18.4
Cognition	76.4	85.0	77.9	79.8	75.5	71.2	69.1	58.1	64.7	37.9	55.1
Psychological capacity	76.0	75.4	81.2	77.2	77.5	70.0	75.0	67.3	67.2	64.3	67.4
Hearing	63.9	78.5	67.0	70.6	57.8	57.4	44.8	40.2	35.7	30.7	37.8
Vision	67.3	73.6	73.9	68.6	68.8	57.4	59.6	43.4	51.9	32.9	52.0
High IC (high capacity in all subdomains)	31.0	40.2	39.8	32.5	29.8	17.5	19.3	4.9	11.1	2.9	1.0
Intermediate IC (low capacity in 1–2 subdomains)	44.7	45.4	44.2	47.4	46.7	45.4	45.9	37.3	39.5	21.4	35.7
Low IC (low capacity in 3–6 subdomains)	24.3	14.4	16.0	20.1	23.5	37.1	34.8	57.8	49.4	75.7	63.3

Locomotor capacity demonstrated the strongest association with self-perceived health, as reported by the estimated odds ratios (ORs) in Model 3, in the logistic regression models. In contrast, cognitive capacity exhibited the weakest association with self-perceived health. All associations remained statistically significant across all three models (Table 3).

**Table 3:** Results from logistic regression for reporting good health by the subdomains of intrinsic capacity.

N = 8718	Model 1 OR (95% CI)	Model 2 OR (95% CI)	Model 3 OR (95% CI)
Vitality	3.54 (3.07 – 4.08)	3.22 (2.78 – 3.72)	2.36 (2.01 – 2.77)
Locomotor capacity	4.62 (4.17 – 5.12)	4.00 (3.57 – 4.47)	3.24 (2.87 – 3.67)
Cognitive capacity	1.93 (1.74 – 2.13)	1.62 (1.46 – 1.80)	1.28 (1.14 – 1.44)
Psychological capacity	4.15 (3.74 – 4.60)	4.04 (3.64 – 4.49)	3.16 (2.82 – 3.54)
Hearing capacity	3.25 (2.95 – 3.56)	2.96 (2.68 – 3.26)	1.90 (1.68 – 2.16)
Visual capacity	3.50 (3.12 – 3.84)	3.10 (2.81 – 3.42)	1.85 (1.63 – 2.09)

Model 1: Unadjusted.

Model 2: Adjusted for age, sex, and education.

Model 3: Adjusted for age, sex, education, and other IC subdomains.

All p-values < 0.001.

### 3.1 Intrinsic capacity and self-perceived health over age and education

Likelihood ratio tests indicated that the three-factor interaction (number of low-capacity IC subdomains × age × education) did not significantly improve model fit compared to models with two-factor interactions (number of low-capacity IC subdomains × age, number of low-capacity IC subdomains × education, and age × education), neither for the total sample (p = 0.460), nor women (p = 0.408), nor men (p = 0.556). The two-way interaction between age and education did not improve the model fit either (total: p = 0.872; women: p = 0.152; men: p = 0.102). Therefore, we present results from models including the two-way interactions between the number of low-capacity IC subdomains and age, and between the number of low-capacity IC subdomains and education.

For the total sample, we observed significant interactions between the number of low-capacity IC subdomains and education (p = 0.005) and between the number of low-capacity IC subdomains and age (p = 0.001). Among women, both interactions were significant (education: p = 0.009; age: p = 0.001), whereas neither interaction was significant for men (education: p = 0.134; age: p = 0.203).

**Fig. 2:**
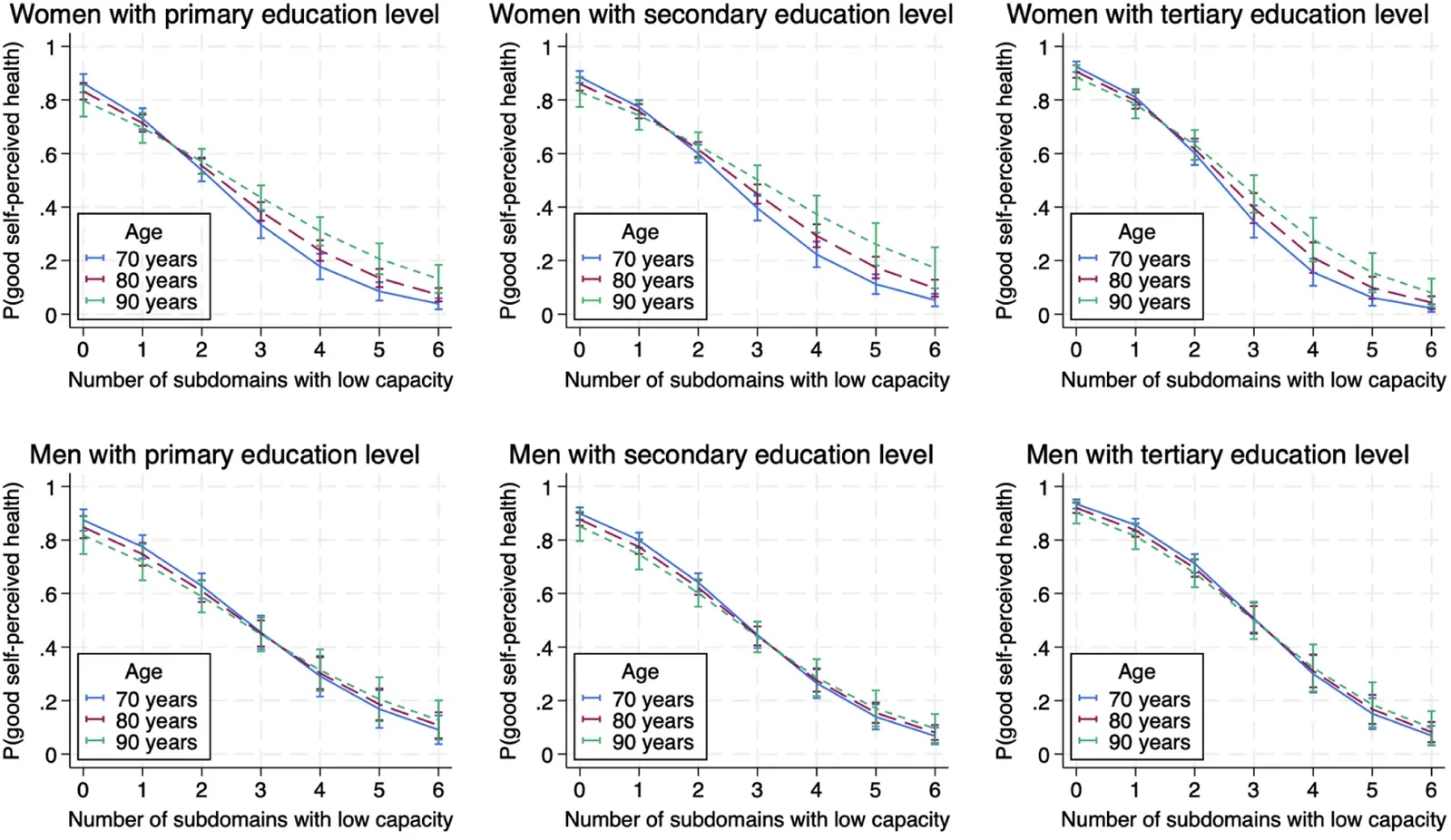
Margins plot of self-perceived health and number of intrinsic capacity subdomains with low capacity, by sex, age group, and education, shown in plots for different education levels. Predicted values with 95% CIs are shown.*.

From the margins plot, the significant interaction term between age and the number of low-capacity IC subdomains in women appears to be caused by an attenuation of the negative effect of more low-capacity IC subdomains on self-perceived health at older ages (Fig. 2). Furthermore, participants with tertiary education (≥ 14 years) seem to exhibit a slightly higher predicted probability of good self-perceived health with low capacity in a few subdomains, but also a steeper decline in these probabilities when the number of low-capacity subdomains increases, compared to those with secondary (11–13 years) or primary (≤ 10 years) education. Furthermore, the oldest women had the flattest curve, suggesting that a higher number of IC domains with low capacity had a smaller impact on their self-perceived health compared to younger women (Fig. 2).

* All margins plots are based on the same model, including the two-factor interaction terms IC subdomains x age, and IC subdomains x education.

## 4 Discussion

In this cross-sectional population-based study, we aimed to describe the distribution of IC across age and sex in a population-based sample of community-dwelling older adults in Norway and examine the associations between IC subdomains and self-perceived health.

We found an association between high capacity and good self-perceived health in all IC subdomains. For each subdomain, we observed a lower percentage of individuals with high capacity in older age groups than in younger age groups. High locomotor capacity was the strongest predictor of good self-perceived health, and the subdomain with the most pronounced difference in proportions between younger and older age groups. Low IC increased the probability of poor self-perceived health, but less among the oldest women and more among highly educated women when low capacity occurred in multiple subdomains.

IC is a dynamic measure of functional ability [4,8], and as self-rated health is recognized as a good indicator of individual health status [29], examining this association can provide valuable insights into how underlying capacities shape perceived health. In 2025, Chu et al. identified age-specific patterns across the IC subdomains, with vitality emerging as the strongest determinant of good health in the oldest adults, locomotor and psychological capacity in young-older adults, and psychological capacity in the middle-aged group, suggesting age-specific health strategies [14]. In our larger, older sample, we did not analyze for any age-specific pattern but identified locomotor capacity as the predominant predictor of self-perceived health. And while this overlaps with the pattern in young-older participants (65 – 74 years old), reported by Chu et al., discrepancies in some of the other findings may stem from differences in sample characteristics, or in IC assessments [14]. Chu et al. provide valuable age-specific insights into IC and self-rated health, and our study complements their work by including a wider span of older adults. We also use clinically assessable tools to evaluate the separate IC subdomains, such as the SPPB rather than a 200- or 300-meter walk to assess physical capacity.

In our analysis, locomotor capacity showed the strongest adjusted association, underscoring its relevance to self-perceived health among older adults. As part of an interdisciplinary approach, monitoring physical function in healthy older adults can facilitate early detection of functional decline and age-related health changes [30]. And while other studies have emphasized vitality as a core domain [11,17], our study shows that locomotor capacity plays an important role in self-perceived health among older adults.

The IC subdomains were previously found to interact closely with one another [31], appearing to be interrelated and functionally inseparable [3,9]. The observed changes in ORs after adjusting for other subdomains were therefore anticipated, with all associations remaining statistically significant. Maintaining high capacity within one subdomain could therefore positively influence other subdomains, reinforcing the holistic nature of IC. As perceived health is central to well-being in older age [32,33], our findings suggest that IC may serve as a meaningful measure of health, consistent with the ICOPE premise that examining IC facilitates early identification of functional decline and informs preventive interventions.

The association between IC and age, sex, and education has been reported previously [9,18]. In our analysis, we further observed that age and education moderated the association between the number of low-capacity IC subdomains and self-perceived health in women, suggesting that sociodemographic factors shape how they perceive their own health. Among the oldest women, low capacity in several IC subdomains appeared to have a smaller impact on self-perceived health than in younger women. We also observed a steeper decline in estimated self-perceived health with an increasing number of low-capacity subdomains among women with tertiary education compared to women with lower levels of education. These observations may reflect differences in expectations concerning health and well-being among older adults and what shapes their perception of health [32,34]. Women with higher education may perceive low capacity as more disruptive than those with lower education, and older women seem to consider low IC as part of aging to a larger degree than younger women.

### 4.1 Strengths and limitations

To our knowledge, this is one of the few studies examining the association between IC and self-perceived health in a large sample of community-dwelling older adults. Our study benefits from a large sample with a high age distribution measured with clinically accessible tools, providing a cohort likely more representative of the oldest segments of the population, who are at greater risk of functional vulnerability and IC decline [13,14]. The broad variation in older age, IC, and self-perceived health strengthens this study`s contribution to understanding health and aging. The large sample size strengthens the statistical power and generalizability within this group.

A key strength of this study is the use of well-established clinically relevant measurements aligned with the ICOPE framework to assess IC [6], which increases clinical relevance of the findings. By applying the framework to a large population-based dataset of older adults, we provide robust insights supporting healthy aging while contributing to the foundation for future research employing standardized IC assessments and important knowledge for health care planners.

The HUNT population and Trøndelag County have been recognized as a good reflection of the Norwegian population [21]. In this study, we chose to exclude participants living in nursing homes as they typically present with high levels of frailty and advanced functional decline, limiting the potential for preventive interventions. Community-dwelling older adults represent a key group for interventions aimed at preserving IC and promoting healthy aging. They also demonstrate a larger potential for maintaining health and functional ability through early preventive strategies than institutionalized individuals. Therefore, our findings can’t be generalized to the nursing home population.

In the present study, vitality, psychological, and sensory capacity were assessed through questionnaires. The WHO ICOPE framework encourages performance-based or clinical measures when feasible, and we recognize that relying solely on subjective measurements is a limitation. Nonetheless, the screening tool described in the ICOPE guidelines initially uses questionnaire items to identify potential deficits, before any objective follow-up is undertaken [6]. In the main HUNT4 examination, these subdomains were assessed only through self-report measures. An audiometric test was offered as an optional sub-study to only a randomly selected subset of participants. Consequently, given the available data and the pragmatic constraints of a large, population-based study, we chose to include the questionnaire-derived subdomain scores.

Participants with missing data were generally older and reported good health less frequently than those included in the analysis, which may bias results toward a younger, healthier sample. Nevertheless, we managed to include a large sample of older adults with wide variation in health and IC, enabling us to capture meaningful patterns across different levels. And while healthier individuals may be slightly overrepresented in our sample, our conclusion remains robust and relevant for understanding these associations in older adults.

In this study, all IC subdomains and self-perceived health were converted into categorical variables for the analysis, which may result in loss of information. However, as many of the IC subdomains have established clinical thresholds, we believe that using these thresholds in the analysis increases clinical relevance.

### 4.2 Clinical implications and future research

This study offers useful cross-sectional associations and shows that higher IC is associated with greater odds of good self-perceived health, suggesting that IC is not merely theoretical but a clinically useful measure reflecting older adults' own health perception. It also supports the value of routinely assessing IC in clinical practice to identify individuals at risk of health decline, even before disease or functional impairments appear. IC may therefore serve as a relevant tool in the shift towards more proactive capacity-oriented approaches in clinical care and research. Given the growing need for evidence-based strategies to promote healthy aging, further research is warranted to understand how IC and self-perceived health can be preserved over time. Because the present study uses a cross-sectional design, it cannot determine the direction of the relationship between IC and self-perceived health, nor can it validate IC as a causal determinant of health perception. Therefore, longitudinal data is needed to establish temporal precedence.

## 5 Conclusion

Our findings reveal a robust association between IC and self-perceived health, demonstrating the relevance of IC in general, with locomotor capacity emerging as the subdomain with the strongest link. Given that the total number of low-capacity IC subdomains is associated with older adults’ perception of health, we propose that IC offers a relevant framework for identifying individuals at risk of health decline. The use of IC can help tailor interventions to optimize functional ability and well-being, and we therefore suggest it should be integrated into clinical research and routine assessment of older adults.

## Ethical approval

This study was approved by the Regional Committees for Medical and Health Research Ethics in Norway (R.E.C) (id. 32333).

## Consent to participate

Participants gave written consent to participate in the HUNT4 Study. If participants, for any reason, were judged to not have the ability to give consent themselves, consent was given by their next-of-kin on the participants' behalf.

## Funding

The Norwegian Fund for Post-Graduate Training in Physiotherapy (Fysiofondet) financed the project “Physical function and frailty within the elderly population in Norway: HUNT4 70+ (id. 118159 to K.N.M), but were not involved in the design of the study, collection, analysis, or interpretation of data. They were neither involved in the preparation of the manuscript nor in the decision to submit it. The Norwegian University of Science and Technology (NTNU) provided funding to open access.

## Data statement

The HUNT data are stored in HUNT databank, and to protect the participants’ privacy, the HUNT Research Centre aims to limit storage of data outside HUNT databank. It therefore cannot be deposited in open repositories. HUNT databank can reproduce precise information on all data exported to this project upon request. For more information, see: http://ntnu.edu/hunt/data.

## Declaration of generative A.I. and A.I.-assisted technologies in the writing process

During the preparation of this work, the authors used Microsoft365 Copilot (Microsoft, 2023) as support in the writing process. After using this tool, the authors reviewed and edited the content as needed and take full responsibility for the content of the published article.

## CRediT authorship contribution statement

**Kjerstin Næss Melsæter:** Writing – review & editing, Writing – original draft, Visualization, Methodology, Formal analysis, Data curation, Conceptualization. **Turid Follestad:** Writing – review & editing, Methodology, Formal analysis. **Gro Gujord Tangen:** Writing – review & editing, Supervision, Conceptualization. **Beatrix Vereijken:** Writing – review & editing, Supervision, Conceptualization. **Pernille Thingstad:** Writing – review & editing, Supervision, Conceptualization.

## Declaration of competing interest

The authors declare that they have no known competing financial interests or personal relationships that could have appeared to influence the work reported in this paper.
